# Clampless In-Situ Immobilized Branching (CLIMB) to Reconstruct the Internal Iliac Artery

**DOI:** 10.3390/life12111928

**Published:** 2022-11-18

**Authors:** Takuro Shirasu, Atsushi Akai, Manabu Motoki, Masaaki Kato

**Affiliations:** 1 Department of Cardiovascular Surgery, Morinomiya Hospital, Osaka 536-0025, Japan; 2 Division of Vascular Surgery, Department of Surgery, The University of Tokyo, Tokyo 113-8655, Japan

**Keywords:** internal iliac artery, in-situ branch, clampless, endovascular, hybrid

## Abstract

Background: Surgical reconstruction of the internal iliac artery (IIA) or its branches is sometimes demanding because of difficulty in distal clamping and suturing in the narrow pelvic space. Here we present a hybrid technique of ClampLess In-situ imMobilized Branching (CLIMB) to reconstruct IIA. Methods: in the CLIMB technique, an 8 mm artificial graft is sutured onto the surface of the common iliac artery (CIA) without clamping. Following puncture of the CIA wall, stent grafts are bridged from IIA to the graft. Finally, the graft is sutured to the ipsilateral external iliac artery (EIA). Concomitant endovascular aneurysm repair or IIA branch embolization can also be performed. We applied this technique to the patients unsuited for other IIA reconstruction. Results: eleven patients underwent the current technique. All but one patient underwent contralateral IIA interruption. Seven patients had a history of aorto-iliac repair before the index surgery. Iliac extender, internal iliac component, Viabahn VBX or Fluency covered stent were used for bridging the graft. Simultaneous IIA branch embolization was performed in 2 patients. Distal landing zones were IIA in 7 grafts, superior gluteal artery in 4 grafts and inferior gluteal artery (IGA) in 1 graft. Technical success was achieved in all cases. No patient complained of buttock claudication or other ischemic symptoms on the treatment side. During a mean follow-up period of 38 months, 11 out of 12 grafts were patent without any related endoleak. One IGA graft occluded at 56 months after surgery. Conclusions: the CLIMB technique is a viable alternative to preserve IIA with an acceptable mid-term durability.

## 1. Introduction

The internal iliac arteries (IIAs) perfuse to the gluteal muscles, pelvic organs, and the spinal cord; thus, sacrifice of antegrade IIA flow can result in buttock claudication, colorectal ischemia, erectile dysfunction, and spinal cord ischemia, leading to severe morbidity or lower quality of life [1,2,3]. At endovascular aneurysm repair (EVAR), common iliac arteries (CIAs) are often aneurysmal and unfit for distal landing; in such cases, extension to external iliac artery is required, and IIAs are either reconstructed or interrupted. While unilateral and bilateral IIA interruption at EVAR causes buttock claudication in 27.2% and 36.5%, respectively, preservation of IIA is associated with buttock claudication in only 4.1% [1]. Other ischemic complications such as colorectal ischemia, erectile dysfunction, and spinal cord ischemia are encountered in less incidences, but it is probable that those ischemic complications will be prevented when the antegrade IIA flow is preserved. Based on the reproducible evidence, preservation of at least one IIA is recommended in the guidelines [4,5].

Revascularization methods to preserve IIA at EVAR include iliac branch devices [6], external to internal iliac artery bypass [3], parallel stent grafts [7], and the bell bottom technique [8]. Of these, iliac branch devices exhibit 95% primary patency of IIA at 5 years and are considered as a preferred option [5]. Although we use iliac branch endoprosthesis (W.L. Gore & Associates, Flagstaff, AZ, USA) as long as the anatomical requirements are fulfilled, the intrinsic device limitations include length from the lower renal artery to ipsilateral iliac bifurcation, CIA diameters or IIA diameters, which can preclude use of iliac branch endoprosthesis in more than half of patients [9]. On the other hand, several reports show that bell bottom technique for ectatic CIA is a risk factor for delayed type 1b endoleak (CIA ≥ 17 mm [10] or use of flared iliac devices ≥ 20 mm [11]), with an estimated incidence of 16% at 5 years. Therefore, some alternatives to iliac branch endoprosthesis are warranted in case outside of instruction for use.

We applied the current hybrid approach of ClampLess In-situ imMobilized Branching (CLIMB) to reconstruct IIAs in such cases as prior EVAR with narrow legs or history of open aneurysm repair. A clampless hybrid in-situ branching reinforced by stent graft for total debranching of supra-aortic trunks (which was named real chimney technique at that time) is reported elsewhere, which demonstrated its feasibility [12]. Since reconstruction of IIA incorporate additional endovascular procedures such as directional branching and branch embolization, this study was intended to present the surgical procedures of the CLIMB technique, specifically to reconstruct IIA, and to report the perioperative and mid-term outcomes. The clampless hybrid in-situ branching reinforced by stent graft was renamed as CLIMB to elude misunderstanding recalled by the term “chimney”, which reminds endovascular interventionists of parallel grafting.

## 2. Patients and Methods

We analyzed the surgical outcomes of patients who underwent IIA reconstruction using the CLIMB technique at Morinomiya Hospital in Japan between 2010 and 2022. Background characteristics of patients were collected including hypertension, diabetes mellitus, dyslipidemia, chronic kidney disease, coronary artery disease and cerebrovascular disease. Chronic kidney disease was considered present when the estimated glomerular filtration rate was below 60 mL/min/1.73 m^2^. Coronary artery disease is defined as either history of coronary artery bypass grafting, percutaneous coronary intervention, or documented evidence of the disease. Cerebrovascular disease has a history of cerebral infarction or cerebral bleeding. Indication for intervention was presented for each case together with prior history of aortoiliac reconstruction and contralateral IIA interruption. Procedural details were also demonstrated regarding stent grafts used for bridging from the distal landing zone and the artificial in-situ branch. Distal landing zones were shown as IIA if the landing zones were proximal to the branching of SGA; otherwise, the names of the artery landed were presented. Concomitant aortic procedures were the procedures at the same operation of the CLIMB technique, and a staged procedure was excluded from this record. Informed consent for presentation of the anonymized data was obtained from each patient.

### 2.1. Perioperative Management

As perioperative management, all patients were screened for their ability to undergo general anesthesia and surgery. Routine checkups include laboratory data, X-rays, electrocardiography, echocardiography, spirometry, ultrasonography on carotid arteries, renal arteries, and abdominal arteries, ankle brachial index, and contrast-enhanced computed tomography. The patients were consulted to a rehabilitation team before operation, and postoperative rehabilitation was started usually on postoperative day 1. The patients were encouraged to walk as much as they can, and presence of buttock claudication was routinely checked on each side. Normal diet was resumed after confirming the recovery of bowel movement based on physical examination, radiographic imaging, and laboratory tests. In case the abnormal findings were noted, such as diarrhea, melena, and abdominal pain, further examination including computed tomography and colonoscopy were considered to investigate presence of colonic ischemia. Regarding diagnosis of spinal cord ischemia, any suspicious symptoms were further scrutinized with prompt consultation to neurologists. Lengths of hospital stays were from the day of admission and the day of discharge; preoperative and postoperative periods can vary if the patients prefer to stay longer. However, patients were allowed to discharge after confirming wound stability and absence of postoperative complications that need urgent intervention, based on physical examination and objective findings of ultrasound or computed tomography. Postoperative surveillance was conducted at 1 month, 3 months, 6 months, 1 year and yearly thereafter. On every visit, X-ray image, ultrasonography and computed tomography were performed to evaluate stent graft-related problems such as endoleak, migration, limb patency, limb stenosis, prosthetic graft infection, thrombosis, embolism, vascular injury, and rupture. Presence or resolution of buttock claudication was also interviewed at every visit.

### 2.2. Surgical Procedures

**Figure 1:**
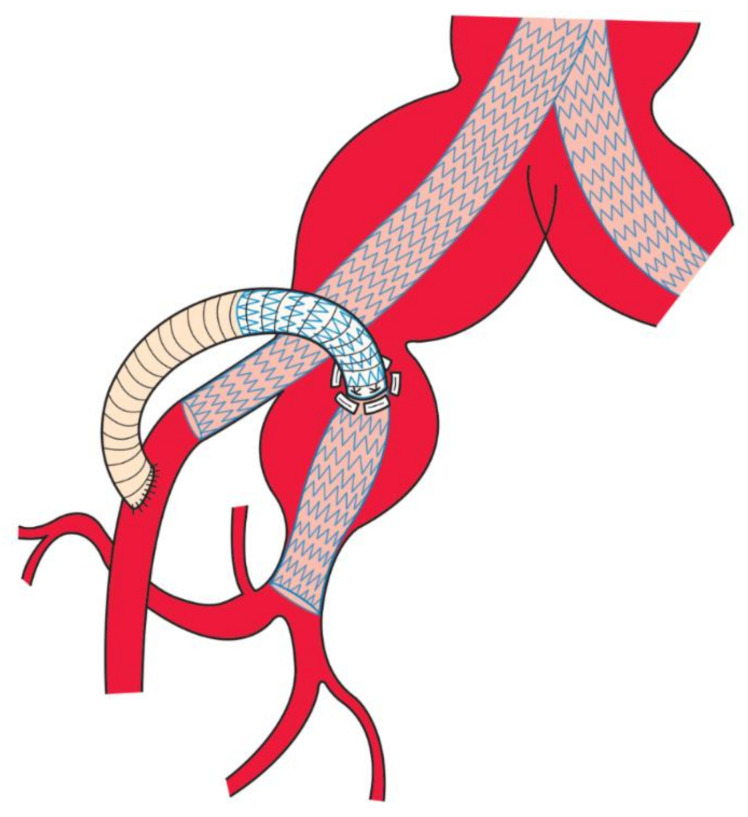
Schematic image of the ClampLess In-situ imMobilized Branching (CLIMB) technique. An artificial graft is sewn to the internal iliac artery (IIA) without clamping. Stent grafts are used to bridge between IIA and the artificial graft. The artificial graft is sewn to the external iliac artery.

The CLIMB technique for IIA begins with a pararectal longitudinal incision. Via a retroperitoneal approach, the surface of the ipsilateral CIA or proximal IIA is dissected. Using 5-0 polypropylene sutures (4–6 separate sutures) with pledgets, a 7–8 mm artificial graft is circumferentially sutured onto the planned puncture site. CIA or proximal IIA is punctured with a percutaneous transhepatic cholangiography drainage needle through the artificial graft, a guidewire is advanced into IIA, and 5-0 polypropylene sutures are tied to avoid leakage. After systemic heparinization (activated clotting time maintained >250 s), a 6Fr sheath is advanced beyond the punctured artery, and the guidewire is changed to a stiff wire such as Amplatz Extra-Stiff wire (Cook Medical LLC, Bloomington, IN, USA). The puncture site is dilated with a 6-mm percutaneous transluminal angioplasty balloon. After confirming backflow from IIA, the artificial graft is filled with heparinized blood, and the artificial graft distal to the puncture site is clamped. Following advancement of 12F sheath into IIA, stent grafts are bridged from IIA to the artificial graft. If multiple stent grafts are required, overlaps longer than 2 cm are recommended to avoid type III endoleak. It is advisable to confirm there is no type 1b or type 3 endoleak while the sheath is still inside the graft. Separate EVAR (either aorto-iliac or iliac EVAR) is performed up to the ipsilateral external iliac artery (EIA). The artificial graft is finally sutured to the EIA distal to the deployed stent graft (Figure 1). The loop of the artificial graft should be designed in a smoothly curved configuration in the retroperitoneal space to avoid kinking and torsion, considering that mobilized peritoneum and organs are settled in the original position after closing the wound. In cases with IIA aneurysms, IIA branches are embolized before insertion of a 12F sheath. After operation, a single antiplatelet agent is administered to maintain graft patency.

**Figure 2:**
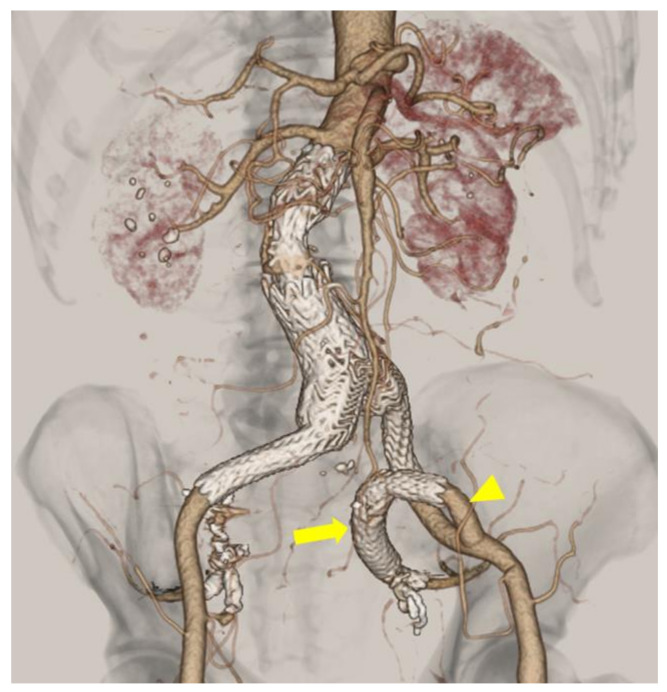
Completion image of the CLIMB technique for IIA. Left IIA reconstruction was performed by the CLIMB technique (patient #8). After embolization of IIA branches, bridging stent grafts (arrow; distal, HGB161007A; proximal, PLC121000) were deployed between the superior gluteal artery and the artificial graft (arrowhead; 8mm Gelsoft Plus, Terumo Corporation, Shibuya, Tokyo, Japan), which was sewn to the external iliac artery. PLC141400J was deployed between the left leg of the prior endograft and the external iliac artery.

A completion image of the CLIMB technique for IIA for patient #8 is shown in Figure 2. This patient had history of EVAR and right IIA embolization before the index left IIAA treatment. After suturing the in-situ branch (8mm Gelsoft Plus, Terumo Corporation, Shibuya, Tokyo, Japan) on the proximal portion of IIA and insertion of a short sheath, IGA was selectively embolized with coils. HGB161007A (distal) and PLC121000 (proximal) were deployed between SGA and the artificial graft which was sewn to the external iliac artery. PLC141400J was deployed between the left leg of the prior endograft and the external iliac artery proximal to the anastomosis.

### 2.3. Tips of the CLIMB Technique

Since the CLIMB technique is a hybrid procedure which requires both open surgical and endovascular skills, there are some technical tips. First, retroperitoneal dissection should be as minimal as possible, and dissection should be performed right along the retroperitoneum, never toward the lateral side. Herein, the psoas muscle is a good landmark. Second, the puncture site on the arterial wall is tailored to each anatomy considering the route inside and outside the artery. Puncture on the artificial graft should be distal to the assumed anastomosis. Third, conflict of the CLIMB graft with external iliac extension was never experienced, but meticulous planning on the route of the stent grafts is advisable. Fourth, in case of additional endovascular procedures such as embolization, 6 sutures with pledgets are optimal to avoid bleeding before reinforcing the anastomosis site by the bridging stent graft. Fifth, the fenestrated wall should be dilated enough with non-compliant balloons.

### 2.4. Advantages of the CLIMB Technique over Other Alternatives at Planning

There are some benefits of the CLIMB technique over open surgery or endovascular alternative techniques. First, it does not require clamping. Internal iliac artery aneurysms (IIAAs) are sometimes dilated to the level of the greater sciatic foramen, therefore securing distal clamping sites is often impossible in open surgery. Second, CLIMB allows simultaneous endovascular procedures such as IIA branch embolization and EVAR. Third, CLIMB can be performed if stent graft bridging from CIA to EIA can be completed. This is in contrast to Gore iliac branch endoprosthesis which cannot be performed in cases of narrow CIAs (e.g., <16 mm), prior EVAR (e.g., <16 mm) or prior open aneurysm repair with narrow legs (open repair rarely uses legs >16 mm), and short aortoiliac aneurysms.

## 3. Results

### 3.1. Characteristics of the Eligible Patients

Eleven patients underwent IIA reconstruction with the CLIMB technique. All patients were male with a mean age of 69 ± 12 years old. Comorbidities were hypertension (91%), dyslipidemia (73%), chronic kidney disease (64%), and cerebrovascular disease (18%). All patients had dilated or dissected CIA unsuitable for distal landing. Eight patients had aneurysms in IIA, and 5 patients bilaterally (Table 1).

### 3.2. Details of Operation Procedures

Operative details are shown in Table 1. The contralateral IIA was interrupted in 10 out of 11 patients. The remaining 1 patient had a history of extensive aortic graft coverage due to aortic dissection, and preservation of IIA was indicated to prevent potential spinal cord ischemia [4]. Either iliac leg or extender, internal iliac component, Viabahn VBX (W.L. Gore & Associates), or Fluency^TM^ stent graft (BD, Franklin Lakes, NJ, USA) were used for reconstruction of IIA. A total of 12 grafts were included for analysis. One patient was treated with separate grafts to superior gluteal artery (SGA) and inferior gluteal artery (IGA). The trunk of IIA was chosen as a distal landing zone in 7 grafts, SGA in 4 grafts, and IGA in 1 graft. Concomitant embolization of IIA branches was performed in 2 cases. Bifurcated EVAR was simultaneously performed in 6 patients; otherwise, iliac EVAR from the ipsilateral CIA to EIA was conducted as a part of the CLIMB technique for IIA. Duration of operation was 338 ± 158 min, and estimated blood loss was 497 ± 403 g.

### 3.3. Postoperative Outcomes

Postoperative outcomes are presented in Table 2. Buttock claudication was never observed on the ipsilateral side (0%). Two patients complained of buttock claudication on the contralateral (embolized) side (18%). However, no other ischemic complications such as erectile dysfunction, colonic ischemia, or spinal cord ischemia were reported. Length of hospitalization was 14 ± 5 days. There was no perioperative death.

During a mean follow-up period of 38 ± 39 months, 11 out of 12 grafts remained patent, with 1 graft to IGA occluded at 56 months after surgery. No branch instability was noted. There were no type 1a, type 1b, or type 3 endoleak, migration, stenosis, or other stent graft related complications. Five out of 11 patients were diagnosed with type 2 endoleak, which was considered not related to the CLIMB technique. There was no new-onset buttock claudication on the ipsilateral side. Four patients died during the follow-up period. Causes of death were pneumoniae (8.9 months), heart failure (64.6 months), colon cancer (101 months), and lung cancer (111.9 months). None of them were related to the index aneurysm repair.

## 4. Discussion

The current study demonstrated operative procedures, perioperative feasibility, and mid-term durability of the CLIMB technique to reconstruct IIA. Given the technical challenge of clamping and anastomosis of distal IIA, especially at the levels of its division branches, and versatility of the CLIMB technique in cases that are unsuitable for manufacture-made iliac branch devices, the CLIMB technique serves as a viable option in cases unfit for other methods such as open aneurysm repair or iliac branch devices. In this case series, despite contralateral IIA embolization and prior history of aortic reconstruction, no patient complained of buttock claudication on the ipsilateral side or other ischemic symptoms, showing the importance of preserving antegrade IIA flow.

Overall patency of CLIMB grafts was great with 100% up to 4 years, although 42% of grafts were targeted at the branches of IIA. Reconstruction of IIA division branches using Gore iliac branch endoprosthesis is reported feasible with a primary patency rate of 98% at 1 year [13]. Taken together, IIA division branches can be the target of distal landing in cases of IIA aneurysms. One possible discussion for the selection of a distal target is that SGA may be preferred as a distal landing than IGA since SGA is in general the largest branch of IIA which perfuse large muscles such as the gluteus maximus, although accumulation of evidence is needed to statistically draw a conclusion. Other techniques to preserve IIA at EVAR are external to iliac artery bypass [3], parallel stent grafts [7], and bell-bottom techniques [8]. External to iliac artery bypass was reported from our institution [3], and others [14,15,16]. The reported patency was 100% from all literature at a mean follow-up of 10.1–17 months. However, this surgical bypass demands distal clamp at IIA or division branches. Therefore, we have devised this CLIMB technique without need for clamping. Parallel stent grafts only have short-term outcomes of endoleak, limb occlusion, and reintervention at 6 months, posing concerns for long-term durability [17]. Bell-bottom technique carries increased risk of type 1b endoleak, especially employed in cases with CIA diameter larger than 20 mm [18,19].

Curved configuration of the CLIMB graft in the confined space seems awful for long term patency, which turned out to be unfounded. We speculate that the reason behind the excellent mid-term results is the usage of flexible stent grafts tailored to each patient’s anatomy; we preferentially used Gore iliac legs or internal iliac components. We used several different types of stent grafts for bridging, including Viabahn VBX and internal iliac components. Similarly favorable outcomes of IIA reconstruction using Viabahn VBX compared with internal iliac components are recently reported [20]. Another technical tip is securing an adequate overlap between the stent grafts (e.g., >2 cm) to avoid a type 3 endoleak. It is also worth noting that the site of puncture and configuration of the artificial graft should be designed beforehand to prevent kinking and torsion. Three-dimensional relationship of the landing zone (on IIA), puncture site (on the surface of CIA or IIA), and anastomosis site (on EIA) can be diverse from case to case, but we will recommend refraining from small radius of curvature when designing the route of the graft. Although we did not experience any troubles related to enteral or ureteral fistula (erosion), meticulous care on neighboring tissues is indispensable for extra-anatomical bypass grafting.

Some characteristics of IIAAs can be explained from embryological points of view. IIA is derived from the axial artery, the first artery to penetrate to the lower limb, and is the upstream of the umbilical artery. [21] Since the bilateral umbilical arteries are maintaining placental circulation, a high load of blood during that period is one of the possible reasons for aneurysmal susceptibility of IIAs, together with genetic predisposition, advanced age, and smoking. Along these lines, aneurysms are typically found in the trunk of IIA, while its division branches, such as SGA and IGA, are usually not dilated. Also, IIAAs often coincide with abdominal aortic aneurysms, CIA aneurysms, and bilateral aneurysm development. This is supported by the evidence that iliac artery aneurysms are seen most frequently in CIAs (89%) followed by IIAs (10%) but rarely in EIAs (1%) [22]. From surgeons’ viewpoints, management of IIAAs can be complex in that simultaneous treatment of abdominal aortic aneurysms, CIA aneurysms and contralateral IIAAs is usually required. Another point that needs attention is that IIAAs extend to the levels of its division branches, which makes surgical reconstruction quite challenging.

A clampless feature of the CLIMB technique is beneficial in IIA reconstruction, especially at the levels of its division branches, where the arteries are penetrating the greater sciatic foramen. Securing a distal clamping site is almost impossible in some cases, in which endovascular technique without clamping can effectively reconstruct the IIA branches. Herein, another advantage of the CLIMB technique is that division branch embolization, an important element of endovascular IIAA treatment, can be performed simultaneously before implantation of stent grafts into the IIA.

Another significance of the CLIMB technique in the era of iliac branch devices is that it can be applied to the narrow CIA or prior grafting (either surgically or endovascularly) unfit for those devices. As we discussed before, IIAAs often coincide with aneurysms in the aorta or CIAs, and hence we often encounter cases in which aorto-iliac aneurysms are already treated while IIAs are left untouched because the IIAs were not indicated for repair at the time of the index procedure. In such cases with prior history of surgical aorto-iliac repair, the legs of the bifurcated graft are usually 8–10 mm in diameter, which is inappropriate for endovascular iliac branch devices. However, the CLIMB technique can handle such cases with the proper application as we have presented. We introduce this CLIMB technique not as the first-line option but as a reserved alternative to supplement the contemporary endovascular and surgical options.

The limitation of the current report is that it is a preliminary case series of the CLIMB technique for IIA, and the accumulation of data is necessary to confirm reproducibility in safety, efficacy, and durability.

## 5. Conclusions

We presented detailed surgical techniques of the CLIMB technique with favorable perioperative and mid-term outcomes up to 4 years. When IIAs were reconstructed with the CLIMB technique, patients did not experience ischemic complication such as buttock claudication on the treatment side. Graft patency was 100% at 4 years, albeit 42% of grafts were targeted at the division branches of IIA. A clampless hybrid procedure of the CLIMB technique can incorporate simultaneous EVAR or IIA branch embolization, which makes this technique quite a useful alternative to reconstruct the IIA or its branches in cases unsuitable for surgical repair or manufacture-made iliac branch devices.

## Figures and Tables

**Table 1:** Operative details of included patients.

Pt No.	Age	Sex	Diagnosis	Contralateral IIA Occlusion	Prior Aortic Procedure	Bridging Stentgraft (Size)	Distal Landing	IIA Branch Embolization	Concomitant Aortic Procedures	Other Procedures	Operative Time, Min	Estimated Blood Loss, mL
1	79	M	Bil CIAA, bil IIAA s/p OAR	+	OAR	Iliac leg (12–100)	IIA	-	EVAR, contralateral IIA embolization	None	370	275
2	75	M	AAA, bil CIAA, bil IIAA	+	-	Iliac leg (12–100)	IIA	-	EVAR, contralateral IIA embolization	None	475	440
3	69	M	AAA, bil CIAA, bil IIAA	+	-	Covered stents (SGA, 10–80; IGA, 8–80)	SGA, IGA	-	EVAR, contralateral IIA embolization	FAA repair	622	520
4	39	M	DAA, Chronic TBAD	-	-	Iliac leg (12–70)	IIA	-	EVAR	None	485	130
5	68	M	AAA, Lt CIAA, bil IIAA	+	-	IIC (10–70)	IIA	-	EVAR, contralateral IIA embolization	PTRA	316	420
6	76	M	AAA, lt CIAA s/p Rt iliac EVAR	+	Iliac EVAR	Iliac leg (12–100)	IIA	-	EVAR	PTRA	262	585
7	80	M	Lt IIAA	+ *	-	VBX ×2 (8L–79)	SGA	- ^†^	None	None	171	310
8	80	M	T1bEL after EVAR, IIAA	+	EVAR	Iliac leg (12–100) + IIC (10–70)	SGA	+	IIA branch embolization	None	181	220
9	67	M	T1bEL after EVAR	+	EVAR	IIC ×2 (10–70)	IIA	-	EVAR	None	166	670
10	56	M	Lt CIAA, IIAA s/p OAR	+	OAR	Iliac leg (10–70) + IIC (10–70)	SGA	+	IIA branch embolization	Graft interposition	479	1610
11	71	M	T1bEL after EVAR	+	EVAR	Iliac leg (12–120) + IIC (12–70)	IIA	-	None	None	196	285

AD, aortic dissection; CAD, coronary artery disease; CIAA, common iliac artery aneurysm; CKD, chronic kidney disease; CVD, cerebrovascular disease; DAA, descending aortic aneurysm; DL, dyslipidemia; DM, diabetes mellitus; EBL, estimated blood loss; EVAR, endovascular aneurysm repair; FAA, femoral artery aneurysm; HT, hypertension; IGA, inferior gluteal artery; IIAA internal iliac artery aneurysm; IIC, internal iliac component; M, male; OAR, open aneurysm repair; PTRA, percutaneous transluminal renal angioplasty; SGA, superior gluteal artery; T1bEL, type 1b endoleak; VBX, Viabahn VBX. * Right IIA was embolized at a staged procedure for right IIAA. ^†^ IIA branches were embolized 1 week before surgery.

**Table 2:** Postoperative outcomes of IIA reconstruction by CLIMB technique.

Pt No.	BC, Ipsilateral	BC, Contralateral	LOS	IIA Patency	Other Complications	Survival	Follow-Up Period, mo
1	-	+	14	+	EL-	Dead, pneumoniae	8.9
2	-	-	14	+	T2EL	Dead, colon cancer	101.0
3	-	-	11	SGA+ IGA- *	T2EL	Dead, lung cancer	111.9
4	-	-	26	+	EL-	Dead, heart failure	64.6
5	-	-	11	+	EL-	Alive	45.2
6	-	-	20	+	T2EL	Alive	13.2
7	-	-	14	+	T2EL	Alive	33.2
8	-	-	8	+	T2EL	Alive	19.8
9	-	-	8	+	EL-	Alive	12.9
10	-	+	9	+	EL-	Alive	4.2
11	-	-	14	+	EL-	Alive	0.6

BC, buttock claudication; EL, endoleak; IGA, inferior gluteal artery; IIA; internal iliac artery M, male; SGA, superior gluteal artery; T2EL, type 2 endoleak. * IGA graft occluded at 56 months after surgery.

## Data Availability

The data analyzed in this study are available from the corresponding author (T.S.) upon reasonable request.
